# Does hydroxyapatite coating have no advantage over porous coating in primary total hip arthroplasty? A meta-analysis

**DOI:** 10.1186/s13018-015-0161-4

**Published:** 2015-01-28

**Authors:** Yun-Lin Chen, Tiao Lin, An Liu, Ming-Min Shi, Bin Hu, Zhong-li Shi, Shi-Gui Yan

**Affiliations:** Department of Orthopaedic Surgery, Second Affiliated Hospital, School of Medicine, Zhejiang University, No.88 Jiefang Road, Hangzhou, 310009 P.R. China

**Keywords:** Hydroxyapatite, Porous, Harris hip score, Survival, Total hip arthroplasty

## Abstract

There are some arguments between the use of hydroxyapatite and porous coating. Some studies have shown that there is no difference between these two coatings in total hip arthroplasty (THA), while several other studies have shown that hydroxyapatite has advantages over the porous one. We have collected the studies in Pubmed, MEDLINE, EMBASE, and the Cochrane library from the earliest possible years to present, with the search strategy of “(HA OR hydroxyapatite) AND ((total hip arthroplasty) OR (total hip replacement)) AND (RCT* OR randomiz* OR control* OR compar* OR trial*)”. The randomized controlled trials and comparative observation trials that evaluated the clinical and radiographic effects between hydroxyapatite coating and porous coating were included. Our main outcome measurements were Harris hip score (HHS) and survival, while the secondary outcome measurements were osteolysis, radiolucent lines, and polyethylene wear. Twelve RCTs and 9 comparative observation trials were included. Hydroxyapatite coating could improve the HHS (*p* < 0.01), reduce the incidence of thigh pain (*p* = 0.01), and reduce the incidence of femoral osteolysis (*p* = 0.01), but hydroxyapatite coating had no advantages on survival (*p* = 0.32), polyethylene wear (*p* = 0.08), and radiolucent lines (*p* = 0.78). Hydroxyapatite coating has shown to have an advantage over porous coating. The HHS and survival was duration-dependent—if given the sufficient duration of follow-up, hydroxyapatite coating would be better than porous coating for the survival. The properties of hydroxyapatite and the implant design had influence on thigh pain incidence, femoral osteolysis, and polyethylene wear. Thickness of 50 to 80 μm and purity larger than 90% increased the thigh pain incidence. Anatomic design had less polyethylene wear.

## Introduction

Total hip arthroplasty (THA) is one of the most wide operations in orthopedic practice [1]. Cement was widely used, but high rates of failure of cemented femoral components in active patients have been reported. Cemented primary THAs showed excellent results in the short-term but deteriorated with time, while uncemented primary THAs were not only satisfactory in the short-term but also tended to improve with time. Moreover, some studies showed that uncemented and cemented THAs had the comparable clinical results during the follow-up of 6 years, which stimulated the development of implant with uncemented fixation. The uncemented THAs with porous coating allowed bone ingrowth to achieve a rigid fixation. But the problem of osteolysis and stress shielding meant that the long-term stability of uncemented THA was still in question [2,3]. Meanwhile, uncemented stem fixation had the shortcoming of thigh pain. To address such problem, bioactive coating has been added to uncemented component to enhance the fixation by osseointegration of implant, of which hydroxyapatite (HA) was the most popular one.

HA coating accelerates bone healing and enhances the biologic fixation of implant due to its biocompatibility and osteoconductive potential. Several studies have shown that it could reduce the migration of HA-coated prosthetic components and have better results and higher survival rate than identical press-fit components [4]. However, other studies have shown that HA particles delaminated from the stem surface may induce osteolysis either by stimulating bone loss or by migration to the joint space producing third-body wear [5]. Concerns have been raised regarding the technique and parameters that were used in applying the coating to the stem as well [6]. Most reviews of clinical results, X-ray findings, and revision rates were unable to find the significant difference between hydroxyapatite and non-hydroxyapatite-coated stems [7-12]. Some studies showed that hydroxyapatite-coated components could ensure earlier return to activity, reduction in thigh pain, and fewer radiolucent lines [13-16].

A previous meta-analysis by Gandhi et al. [17] based on nine studies including 1,764 samples showed that survival from aseptic loosening had no difference between the two groups at a mean follow-up of 6.5 years, and the mean Harris hip score (HHS) between these groups demonstrated the same. Another meta-analysis by Goosen et al. [18] with eight RCTs including 857 samples reported the clinical and radiographic results, and there was no difference in HHS, endosteal bone ingrowth, and radioactive lines in the surface area of the prosthesis.

The former analysis only included four RCTs of nine studies, and one of which did not show clear HHS data, and the HA stem was grit-blasted, while porous stem was not. The latter analysis did not target new studies published in the later years. It is essential to update the previous results based on the following reasons. (1) The up-to-date 12 RCTs and comparative studies enlarged the sample size to 9,860 and expanded the population distribution. (2) Compared to the average follow-up of 5.4 years in the previous analysis, the longer 7.5 years of the new analysis may lighten on the long-term efficacy of HA coating for HHS, survival, or radiolucent lines. (3) Insufficient data in the previous meta-analysis led to the incomprehensive evaluation of the potential influential factors on HA coating effects, including the thickness and purity of HA coating, the implant design, and the duration of follow-up. The present analysis updated the meta-analysis on the effects of HA-coated stems on clinical and radiographic results, such as HHS, survival, and thigh pain incidence.

## Methods

Electronic databases were searched with the limited language of English. The result was last updated on Dec. 17, 2012. The search used the following term and Boolean operators: “(HA OR hydroxyapatite) AND ((total hip arthroplasty) OR (total hip replacement)) AND (RCT* OR randomiz* OR control* OR compar* OR trial*)”. The reference lists of all the selected articles were hand-searched for any additional trials. If necessary, we contacted authors to collect additional information.

The trials was included if (1) the patients had trauma, arthritis, or other diseases requiring total hip arthroplasty with age not less than 18, (2) the inclusion of the comparison between a proximally HA/porous coated femoral and a proximal porous coated stem who underwent primary uncemented total hip arthroplasty, (3) the measurement of outcome was at least by one of the following clinical and radiographic results, including Harris hip score, survival, thing pain incidence, radiolucent lines, femoral osteolysis, and polyethylene wear, and (4) they were published randomized controlled trials or comparison observational studies. Trials were excluded if (1) the patients underwent cemented THAs or revision uncemented THAs, (2) the trials were phase I or case report or review or animal models, or only the abstract was available, (3) the coating was only on the cup not the stem, (4) the follow-up was less than 1 year, and (5) they were sub-analysis of previously published meta-analyses.

Two of us (CYL and SMM) independently assessed each trial with a 12-item scale [19], assessing factors such as randomization, allocation concealment, and blindness. We resolved disagreements through discussion.

For each eligible trial, we extracted relevant data and checked the accuracy. In instances of unreported standard error for a mean difference in HHS, we calculated the standard error by converting the *p* value to a z-score and solving for the standard error with the formula: *z* = mean difference/standard error [20]. If the article did not have a certain *p* value and only a range, we just excluded the data [13,21,22]. For the trials [13], which had more than one intervention group with different ranges of HA coating, we combined group B and group C into one intervention group. For the trials [23,24], in which some identical patients were included but with different durations of follow-up, we included both of them to evaluate HHS.

Our main outcome measurements were mean postoperative HHS and the survival of prosthesis from aseptic loosening. We also assessed the incidence of thigh pain and radiographic results. To improve the clinical relevance, we used the weighed mean difference (WMD) and then estimated the relative difference in the change from baseline as the absolute benefit divided by the mean of all the baseline means of the control groups. With the fixed effect model, WMD and 95% confidence intervals (95% CI) were calculated and the data pooling was done using Review Manager 5.1.7.

We calculated the statistical heterogeneity using a *χ*^2^ test on *N* − 1 degrees of freedom (*N* = sample size), with significance at 0.05. We also assessed the inconsistency *I*^2^ using the formula [(*Q* − df)/*Q*] × 100% (*Q* = the *χ*^2^ statistic, *df* = degree of freedom) to describe the percentage of the variability in effect estimates due to the heterogeneity [19]. We considered *I*^2^ value of 25%, 50%, and 75% as low, medium, and high heterogeneity, respectively. A fixed effects model was used if there was no statistical heterogeneity among the studies; otherwise, we used the random effects model.

We developed several subgroup analyses not only to explain the heterogeneity but also to identify the factors potentially influencing the clinical and radiographic results, such as the design of study, thickness, and purity of hydroxyapatite, follow-up duration, and implant type. Because there was revalent data showing that 50 to 80 μm was the acceptable standard [25,26], 50 to 80 μm was chosen to be the interval of HA thickness. Six-year duration was chosen to be the cutoff follow-up duration as there was sufficient data available allowing for the subgroup analysis. Sensitivity analysis was performed through omitting trials to assess the changes in overall effect. Funnel plots were used to assess publication bias among the included trials graphically. Bias can be seen if the plots were widely skewed versus a plot resembling an inverted triangle which represents no bias [27].

## Results

**Figure 1:**
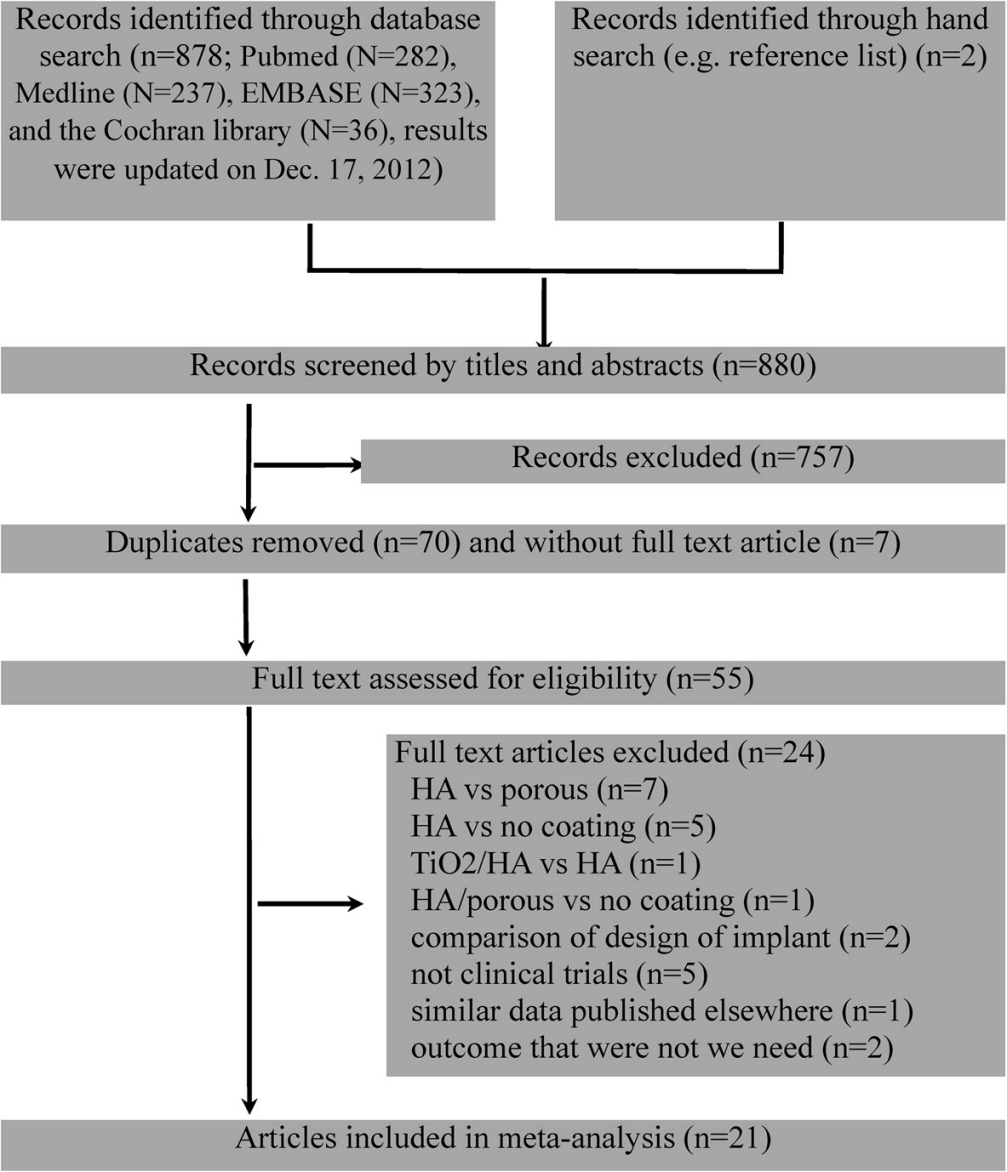
**A flowchart illustrated the selection process of eligible trials in our meta-analysis.**

**Figure 2:**
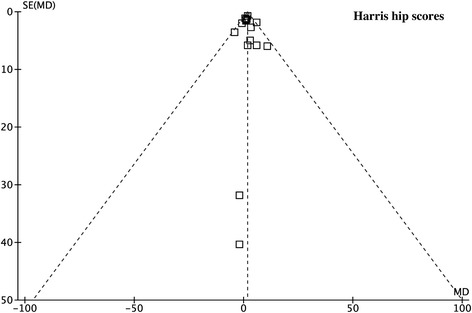
**Funnel plot for HHS shows no publication bias.**

The literature search initially yielded 878 relevant trials. Two of us (CYL and SMM) reviewed the titles and abstracts of all reviews including two of hand-searched. Twelve RCTs [4,22-24,28-35] and nine comparative observation trials [9,10,12,14,16,36-39] were included after applying our eligibility criteria (Figure 1). Funnel plots indicated no publication bias (Figure 2). We recorded the characteristics of 21 trials that were included (Table 1) and details of co-factors and measurement (Table 2). All studies reported a minimum 1 year (median 7.5 years, range 1 to 17.7). We assessed the quality of included trials with the 12-item scale (Table 3). However the intention-to-treat analysis was rarely reported, and no outcome was selectively reported in all studies.

**Table 1:** **Study characteristics**

**Study**	**Study design**	**Sample size (HA/control)**	**Mean ages of patients (years)**	**Sex distribution (female/male)**	**Inclusion criteria**
Camazzola et al. [28]	RCT	61 (34/27)	48.2 ± 9.0/50.4 ± 8.7	22/39	Men younger than 60 years and women younger than 65 years having elective primary THA
Dorr et al. [16]	Retrospective, matched pair	30 (15/15)	55 (38–71)	10/5	Patients who underwent bilateral primary THA
Hamadouche et al. [34]	RCT	45 (22/23)	65/64	41/39	Patients with osteoarthritis of the hip requiring THA
Incavo et al. [32]	RCT	50 (24/26)	55	NR	Patients who underwent THA
Kim et al. [23]	RCT	100 (50/50)	45.3 (27–61)	14/36	Patients who underwent sequential bilateral primary THA
Kim et al. [24]	RCT	110 (55/55)	46.3 (27–63)	39/16	Patients who underwent bilateral primary THA
Lee and Lee 2007 [33]	RCT	40 (20/20)	44 (25–72)	2/18	Patients who had late-stage bilateral osteonecrosis were randomly treated with bilateral THA
Lombardi Jr. et al. [12]	Retrospective, observational study	131 (46/85)	52 (29–72)/51 (22–78)	67/97	Patients who underwent THA using a MHP
Mcpherson et al. [36]	Retrospective, matched pair	84 (42/42)	55 ± 11.4/56.5 ± 11.7	36/48	Patients of the same gender, bone type, activity level, and diagnosis, ages within 5 years, weight within 25 pounds, Charnley activity class
Parvizi et al. [9]	Prospective, matched-pair	86 (43/43)	66.8 ± 6.2/65.7 ± 5.9	NR	The patients matched for age, sex, weight, diagnosis, Charnley class, operative approach, bone quality, femoral head size, type of acetabular component, and duration of follow-up
Paulsen et al. [38]	Retrospective comparison	3,158/4,749	NR	3,834/4,073	Patients underwent primary uncemented THA, who were younger than 70 years of age at surgery
Ranawat et al. [35]	RCT	174 (92/82)	54.9 (29.4–67.5)/55.5 (28.6–71.8)	38/114	Patients received cementless THA with the Ranawat-Burstein metaphyseal-diaphyseal fit hip system
Rothman et al. [10]	Retrospective, matched pair	104 (52/52)	64 (31.2–86.1)	49/49	Consecutive THA with use of Taperloc stem, matched for age, sex, weight, diagnosis, Charnley class, operative approach, and duration of follow-up
Sanchez-Sotelo et al. [37]	Retrospective, matched-pair	136 (68/68)	54 (23–66)/56 (22–67)	56/80	Patients who had a primary hip replacement with insertion of either a porous-coated or HA-coated Omniflex femoral componene
Sano et al. [39]	Retrospective, observational study	55 (24/31)	64.0 (51–83)/62.7 (41–80)	49/3	Patients in whom surgery was performed at least 2 years before the present study
Santori et al. [14]	Retrospective, observational study	227 (158/69)	NR	NR	Patients underwent THA with the anatomic prosthesis
Søballe et al. [4]	RCT	26 (14/12)	56.8 (48–63)/58.6 (50–68)	NR	Patients who underwent THR to receive prosthetic with either Ti-alloy coating or HA coating
Tanzer et al. [22]	RCT	39 (17/22)	66 (54–80)/64 (43–78)	13/26	Patients undergoing a cementless THA
Tanzer et al. [31]	RCT	318 (164/154)	64.5 ± 9.9/63.1 ± 10.5	153/165	All patients who underwent cementless THA
Yee et al. [30]	RCT	62 (35/27)	48.2 ± 9.0/50.4 ± 8.7	11/22	Men younger than 60 years of age and women younger than 65 years of age undergoing primary THA
Yoon et al. [29]	RCT	75 (37/38)	45.3 (20–69)/46.0 (23–71)	14/49	Patients who underwent THA use a multilock femoral stem with or without HA/TCP coating

*RCT* randomized controlled trials, *HA* hydroxyapatite, *NR* not reported, *THA* total hip arthroplasty, *TCP* tricalcium phosphate.

**Table 2:** **Details of co-factors and measurement of studies**

**Study**	**Purity and thickness of HA**	**Surgery approach**	**Co-factors**	**Follow-up (years)**	**Missing information**	**Hip implant**	**Outcome measurement**
Camazzola et al. [28]	NR	Hardinge approach	Routine antibiotic prophylaxis; anticoagulation with dicumarin was used preoperatively and for a total of 3 months postoperatively, full weight-bearing for 6 weeks	13 years and 5 months (12–15 years and 3 months)	4 patients were lost to follow-up, 8 died. 1 refuced to participate in the questionnaire or clinical follow-up	Mallory-Head porous stem (Biomet)	HHS, radiographic outcome, thigh pain, survival
Dorr et al. [16]	94% purity, 50–60 μm	NR	NR	6.5 (5–7.9)	No	Anatomic porous replacement—I hip stem (Intermedics Ortho)	HHS, radiographic outcome
Hamadouche et al. [34]	100 ± 30 μm	Posterolateral approach and a Hardinge lateral approach	Postoperative management include administration of systemic antibiotics for 48 h, preventative anticoagulation therapy until full weight-bearing, and NSAID for 5 days to prevent heterotopic ossification, partial weight-bearing was allowed for 6 weeks followed by full weight-bearing	9.18 (3.93–10.28)	One patient from each group died from an unrelated cause at three months and at 27 months after surgery, three patients were lost to follow-up at a mean of three years, of these, one belonged to the HA and two to the GB group	Profile (DePuy)	HHS, radiographic outcome
Incavo et al. [32]	NR	NR	NR	4	no	ProWle (DePuy)	HHS, radiographic outcome
Kim et al. [23]	30 μm	Posterolateral approach	Stand on the second postoperative day. Partial weight-bearing with crutches as tolerated, full weight-bearing was allowed at 6 weeks after surgery	6.6 (5–7)	No	Cementless IPS femoral component (DePuy)	HHS, functional outcome, thigh pain, radiographic outcome, survivorship, complications
Kim et al. [24]	30 μm	Posterolateral approach	NR	15.6 (15–16)	3 were lost to follow-up, 2 died	IPS femoral stem (DePuy)	HHS, WOMAC, thigh pain, Los Angeles activity score, survivorship
Lee and Lee 2007 [33]	98% purity, 150–250 μm	Direct lateral approach (transgluteal approach)	Hip joint motion and ambulation using a wheelchair were allowed from the first postoperative week, crutch walking with partial weight-bearing began 3 to 4 weeks after the second operation. In addition, the patients used a cane for additional 2 to 4 months until they could walk well without any support	143 (123–168)	4 patients died, 5 patients were lost	Spotorno (Zimmer)	Merle d’Aubigne and Postel score, radiographic outcome
Lombardi, Jr. et al. [12]	95% purity, 50–75 μm	NR	Routine clinical evaluation was performed under the supervision of the operative surgeons	14.5 (10.2–16.6)/16.9 (11.4–18.5)	27 patients in the MHP group and 5 patients in the MHP HA group were decreased from causes unrelated to the index surgery. In addition, 14 patients (14 hips, 10.8%) in the MHP group and nine patients (10 hips, 16.4%) in the MHP HA group had not returned for minimum 10-year follow-up and were lost to contact	Mallory-Head porous stem (Biomet)	HHS, radiographic findings, survival
Mcpherson et al. [36]	94% purity, 50–60 μm	NR	NR	3	No	Anatomic porous replacement—I hip stem (intermedics orthopedics)	HHS, radiographic fixation scores
Parvizi et al. [9]	NR	NR	NR	9.2 ± 4.8/10.1 ± 4.6	Each one of the members of nine pairs of patients is dead	Taperloc, Biomet, Warsaw, Indiana	HHS, radiographic findings
Paulsen et al. [38]	95–97% purity, 50–75 μm	NR	NR	3.2	No	Biometric (Biomet)	Time to implant failure
Ranawat et al. [35]	95% purity, 50–75 μm	Posterolateral approach	Standard postoperative rehabilitation protocol	5 (3–8) for thigh pain, 17.7 + −0.8 (16.3–20)	53 patients were deceased, 28 patients were lost to follow-up	Ranawat-Burstein metaphyseal-diaphyseal fit femoral stem (Biomet)	HSS, functional outcome(patient assessment questionnaire), incidence of pain, radiographic outcome, stem subsidence Kaplan-Meier survivorship
Rothman et al. [10]	95% purity, 50–75 μm	NR	Prophylactic antibiotics were given intravenously at the time of the operation and were continued for 48 h. Ten milligrams of low-dose warfarin was given on the night of the operation, instructed to bear only 10% of the body weight on the affected limb for 6 weeks, at which time, they progressed to use of a cane	2.2 (2–3.4)	No	Taperloc stem (Biomet)	Charnley scores, radiographic outcome
Sanchez-Sotelo et al. [37]	NR	NR	NR	6.7 (2.4–9.1)/9.3 (2.2–11.4)	No	Omniflex stem (Osteonics Corporation)	HHS, radiographs, survival
Sano et al. [39]	NR	Posterior approach	Partial weight-bearing was allowed 1 week after the operation, with full weight-bearing after 3 weeks	34/52 m	No	Biomet (Warsaw); Stryker(Fairfield Rd)	HHS, BMD, radiographic outcome
Santori et al. [14]	70% purity, 80–130 μm	NR	Partial weight bearing with two canes was allowed on the fifth postoperative day and progressed to one cane on day 30	70 m (60–84)	No	Anatomic prosthesis (Zimmer)	HHS, thigh pain, radiographic evaluation
Søballe et al. [4]	50–75 μm	Posterolateral approach	Prophylactic antibiotics and anti-thromboembolic drugs, mobilized on the third postoperative day and instructed to walk with protected weight-bearing for the first six postoperative weeks	1	11 patients were excluded from RSA because of technical errors, 1 patient with bilateral THR died from unrelated disease	Biometric (Biomet)	HHS, the visual analog scale score, radiographs data, RSA
Tanzer et al. [22]	80% HA, 20% TCP, 80 μm	Posterolateral approach	All patients remained non-weight-bearing for 6 weeks, followed by progressive weight-bearing as tolerated	2	No	Cementless multilock stem (Zimmer)	HHS, periprosthetic BMD measurement
Tanzer et al. [31]	80% HA, 20% TCP, 80 μm	Posterolateral approach for 64% and 69% in groups uncoated and coated, lateral approach was used in the remainder	All patients were non-weight-bearing for 6 weeks postoperatively, followed by progressive weight-bearing as tolerated	37 m (2–5 years)	16 patients in the group with uncoated and 11 patients in the group with coated components withdraw or were lost to follow-up; 4 in uncoated and 3 in coated died	Cementless multilock stem (Zimmer)	HHS, WOMAC, radiographic data
Yee et al. [30]	95% purity, 50–70 μm	A modified lateral Hardinge approach	Routine prophylactic antibiotic(cefazolin sodium) was administered before surgery and 48 h after surgery; anticoagulation with dicumarin was given: 5 mg orally the night before surgery and daily for a duration of 3 months after surgery. Physical therapy was commenced on the first or second day after surgery. Tough weight-bearing with crutches for 6 weeks was allowed for uncomplicated cases. Progression to full weight-bearing as tolerated was allowed after 6 weeks	4.6 (3–7)	6 patients were lost to follow-up, 1 died of cardiac causes.1 patient declined additional participation in the study after surgery, 1 with bilateral THA was involved in a motor vehicle accident that resulted in a periprosthetic fracture of one hip	Mallory-Head porous femoral stem (Biomet)	HHS, routine radiographs, survivorship
Yoon et al. [29]	70% HA, 30% TCP, 70 μm	Hardinge’s lateral approach	Instruted to walk with partial weight-bearing with the aid of 2 crutches for 4 weeks after surgery	127.4 m (96–144)/127 (108–144)	2 patients in the coated group died of myocardial infarction and cerebral infarction, 2 patients in coated and 2 in uncoated were lost to follow-up	Multilock femoral stem (Zimmer)	HHS, radiographic evaluation, thigh pain

*HA* hydroxyapatite, *TCP* tricalcium phosphate, *NR* not reported, *HHS* Harris hip score, *WOMAC* Western Ontario and McMaster Universities Osteoarthritis Index scores, *RSA* Roentgen stereophotogrammetric analysis, *HSS* hospital for special surgery hip score.

**Table 3:** **Methodologic quality of included studies**

**Study**	**Randomized adequately** ^**a**^	**Allocation concealed**	**Similar baseline**	**Patient blinded**	**Care provider blinded**	**Outcome assessor blinded**	**Avoid selective reporting**	**Similar or avoided cofactors**	**Patients’ compliance** ^**b**^	**Acceptable drop-out rate** ^**c**^	**Similar timing**	**ITT analysis** ^**d**^	**Quality** ^**e**^
Hamadouche et al. [34]	Yes	Unclear	Yes	Unclear	Unclear	Yes	Yes	Yes	Yes	Yes	Yes	No	High
Incavo et al. [32]	Yes	Unclear	Yes	Unclear	Unclear	Unclear	Yes	Yes	Yes	Yes	Yes	No	High
Kim et al. [23]	Yes	Yes	Yes	Unclear	Unclear	Unclear	Yes	Yes	Yes	Yes	Yes	No	High
Kim et al. [24]	Yes	Unclear	Yes	Yes	Unclear	Yes	Yes	Yes	Yes	Yes	Yes	No	High
Lee and Lee [33]	Yes	Unclear	Yes	Unclear	Unclear	Unclear	Yes	Yes	Yes	Yes	Yes	No	High
Søballe et al. [4]	Yes	Yes	Yes	Unclear	Unclear	Yes	Yes	Yes	Yes	Yes	Yes	No	High
Tanzer et al. [22]	Yes	Yes	Yes	Unclear	Unclear	Unclear	Yes	Yes	Yes	Yes	Yes	No	High
Tanzer et al. [31]	Yes	Yes	Yes	Yes	Yes	Yes	Yes	Yes	Yes	Yes	Yes	No	High
Yee et al. [30]	Yes	Unclear	Yes	Unclear	Unclear	Yes	Yes	Yes	Yes	Yes	Yes	No	High
Yoon et al. [29]	Yes	Yes	Yes	Unclear	Unclear	Unclear	Yes	Yes	Yes	Yes	Yes	No	High
Camazzola et al. [28]	Yes	Unclear	Yes	Unclear	Unclear	Yes	Yes	Yes	Yes	No	Yes	No	Moderate
Dorr et al. [16]	Unclear	Unclear	Yes	Unclear	Unclear	Unclear	Yes	Yes	Yes	Unclear	Yes	Unclear	Moderate
Lombardi, Jr. et al. [12]	Unclear	Unclear	Yes	Unclear	Unclear	Unclear	Yes	Yes	Yes	No	Yes	No	Moderate
Mcpherson et al. [36]	Unclear	Unclear	Yes	Yes	Unclear	Unclear	Yes	Yes	Yes	Yes	Yes	No	Moderate
Parvizi et al. [9]	Unclear	Unclear	Yes	Unclear	Unclear	Unclear	Yes	Yes	Yes	Yes	Yes	No	Moderate
Paulsen et al. [38]	Unclear	Unclear	Yes	Unclear	Unclear	Unclear	Yes	Yes	Yes	Yes	Yes	No	Moderate
Ranawat et al. [35]	Yes	Unclear	Yes	Unclear	Unclear	Unclear	Yes	Yes	Yes	No	Yes	No	Moderate
Rothman et al. [10]	No	Unclear	Yes	No	No	Yes	Yes	Yes	Yes	Yes	Yes	No	Moderate
Sanchez-Sotelo et al. [37]	Unclear	Unclear	Yes	Unclear	Unclear	Unclear	Yes	Yesc	Yes	Yes	Yes	No	Moderate
Sano et al. [39]	Unclear	Unclear	Yes	Unclear	Unclear	Unclear	Yes	Yes	Yes	Yes	Yes	No	Moderate
Santori et al. [14]	Unclear	Unclear	Yes	Unclear	Unclear	Unclear	Yes	Yes	Yes	Yes	Yes	No	Moderate

^a^Only if the method of sequence generated was explicitly described could get a “Yes”; sequence generated by “Dates of Admission” or “Patients Number” received a “No”.

^b^Intermittent treatment or therapy duration less than 6 months means “Yes”, otherwise “No”.

^c^Drop-out rate ≥ 20% means “No”, otherwise “Yes”.

^d^ *ITT* intention-to-treat, only if all randomized patients are analyzed in the group they were allocated to could receive a “Yes”.

^e^The frequency of “Yes” as 7 or greater means “High”, greater than 4 but less means “Moderate”, 4 or less means “Low”.

**Figure 3:**
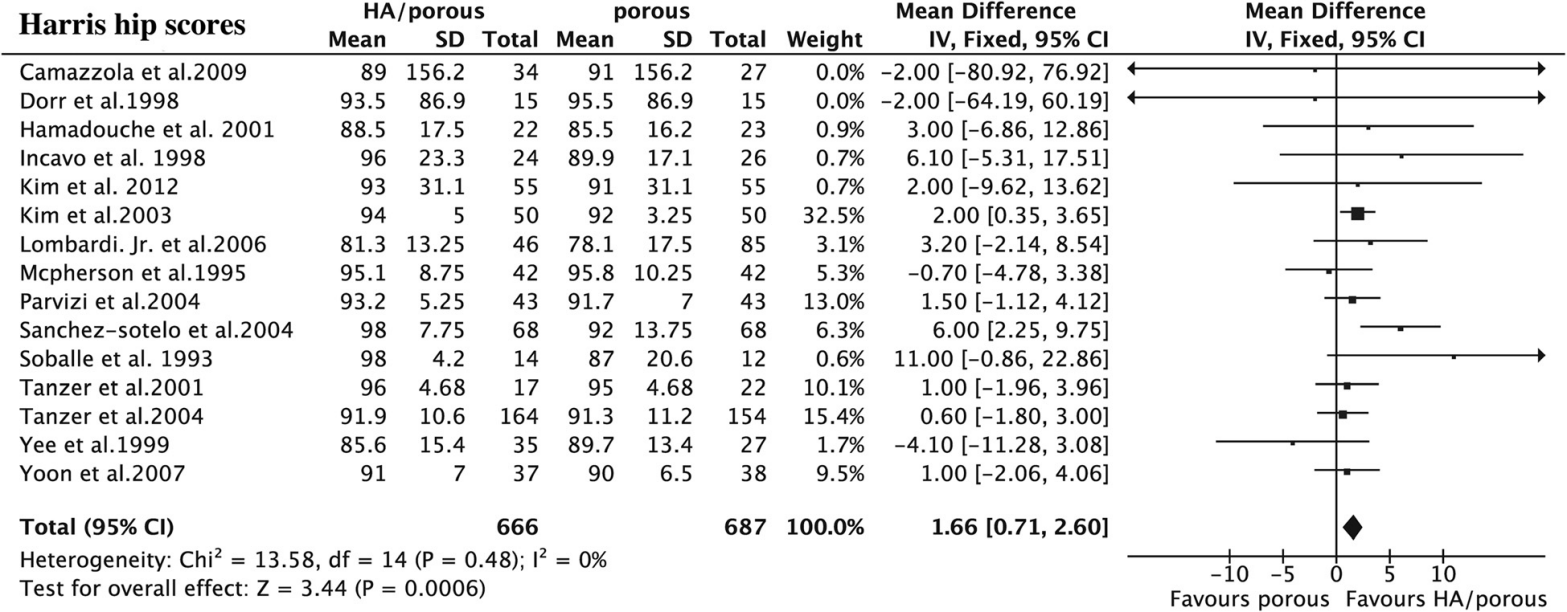
**The forest plot for Harris hip score shows HA coating can improve the post-operative HHS compared with porous coating.** *IV* inverse variance, *HA* hydroxyapatite.

**Figure 4:**
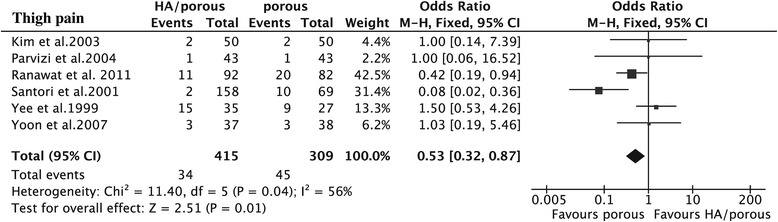
**The forest plot for thigh pain incidence shows that HA coating can reduce it compared with porous coating.** *HA* hydroxyapatite.

**Figure 5:**
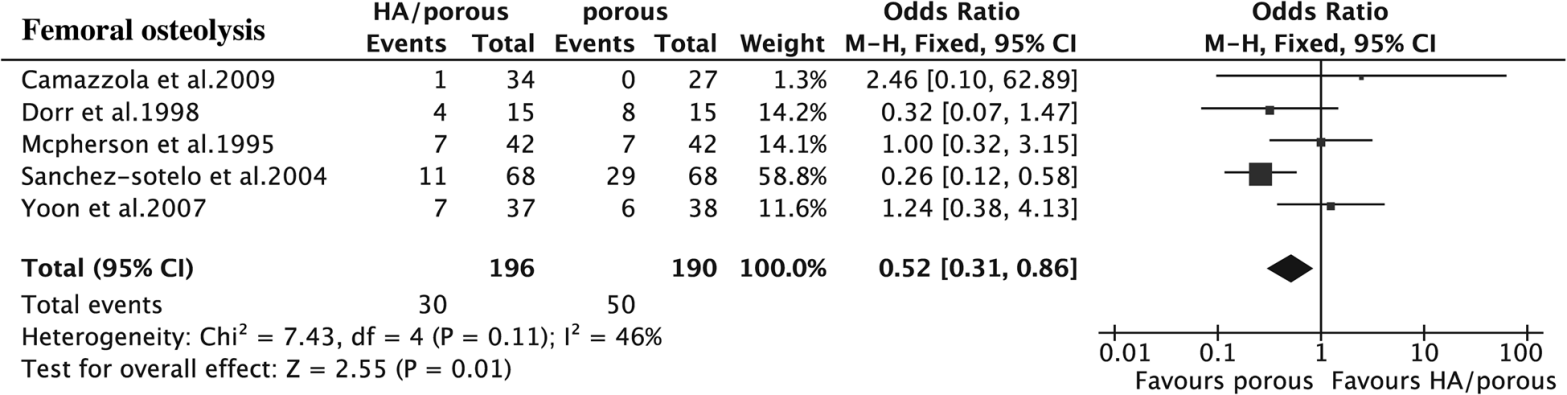
**The forest plot for femoral osteolysis shows that HA coating has less osteolysis compared with porous coating.** *HA* hydroxyapatite.

**Figure 6:**
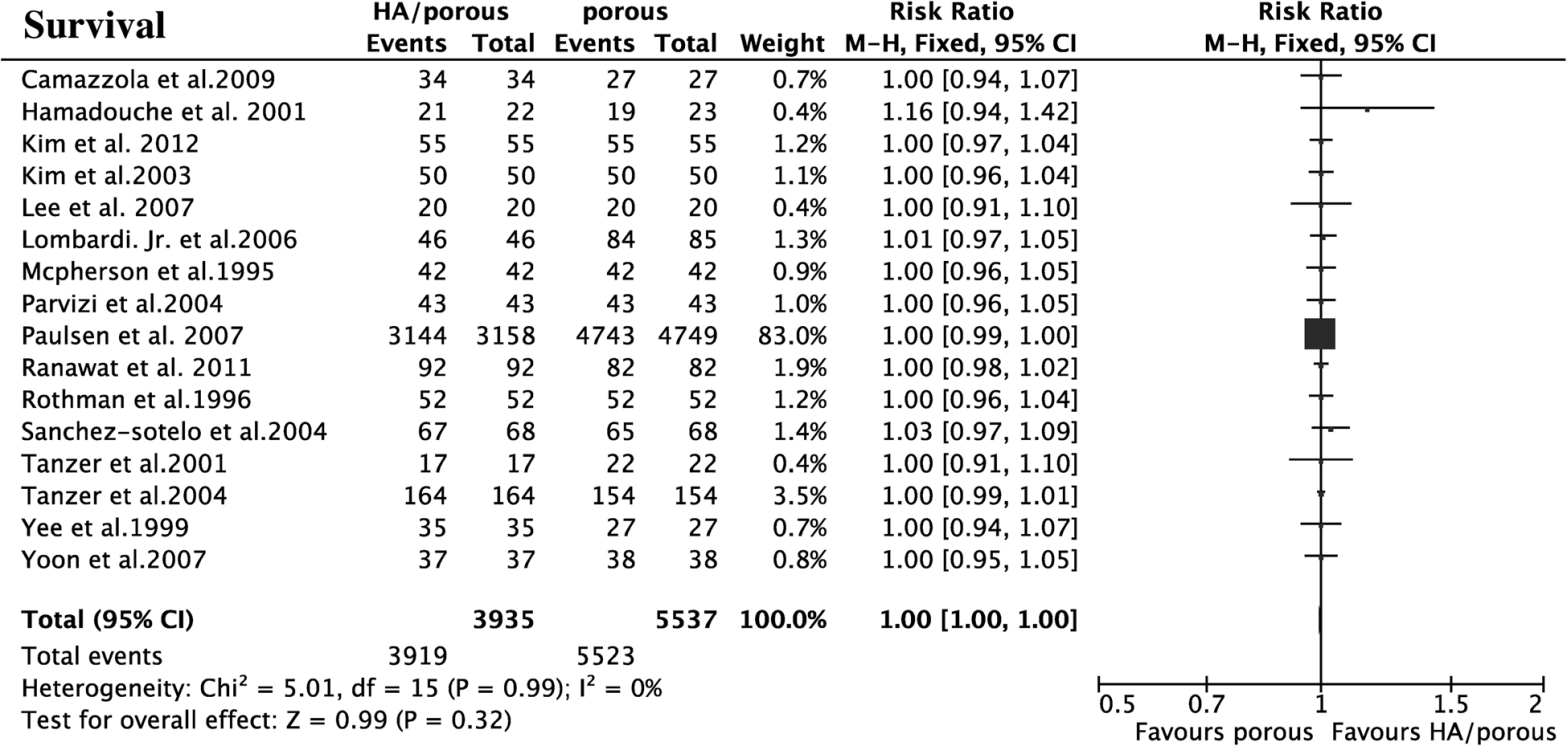
**The forest plot for survival from aseptic loosening shows no difference between HA coating and porous coating.** *HA* hydroxyapatite.

**Figure 7:**
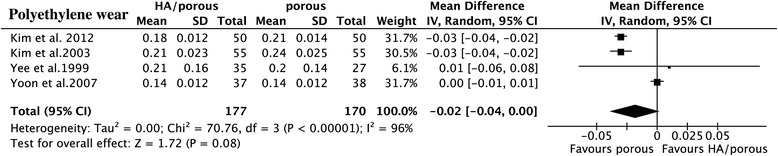
**The forest plot for polyethylene wear shows HA coating has less wear compared with porous coating.** *IV* inverse variance, *HA* hydroxyapatite.

**Figure 8:**
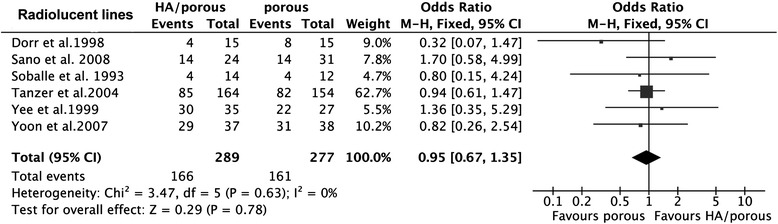
**The forest plot for radiolucent lines shows no difference between HA coating and porous coating.** *HA* hydroxyapatite.

The results showed that the HA presented higher HHS than the porous group (15 trials, *N* = 1,353, WMD = 1.66, 95% CI 0.71 to 2.60, *p* = 0.0006) (Figure 3), could decrease the thigh pain incidence (6 trials, *N* = 724, OR = 0.53, 95% CI 0.32 to 0.87, *p* = 0.01) (Figure 4), and had less femoral osteolysis (5 trials, *N* = 386, OR = 0.52, 95% CI 0.31 to 0.86, *p* = 0.01) (Figure 5), while there was no difference in the survivorship from aseptic loosening (16 trials, *N* = 9,472, RR = 1.00, 95% CI 1.00 to 1.00, *p* = 0.32) (Figure 6), polyethylene wear (4 trials, *N* = 347, WMD = −0.02, 95% CI −0.04 to 0.00, *p* = 0.08) (Figure 7) and radiolucent lines (6 trials, *N* = 566, OR = 0.95, 95% CI 0.67 to 1.35, *p* = 0.78) ( Figure 8) between the two groups.

There were no heterogeneities for HHS (*I*^2^ = 0%), survivorship from the aseptic loosening (*I*^2^ = 0%), radiolucent lines (*I*^2^ = 0%), or femoral osteolysis (*I*^2^ = 46%). The results of thigh pain incidence and polyethylene wear was heterogeneous in some degree (*I*^2^ = 56%; *I*^2^ = 96%). The heterogeneity of thigh pain incidence can not be explained by the study design, quality of study, thickness of HA, implant design or follow-up duration maybe because of the potential co-factors, such as weight-bearing after the operation. As to the limitation of this analysis, we could not set this subgroup analysis. The polyethylene wear can not be explained by purity of HA and duration of follow-up. But when we classified the polyethylene wear into subgroups by the thickness of HA (50–80 μm and >80 μm or < 50 μm) and implant design (anatomic and non-anatomic), the heterogeneity could no longer be observed (*I*^2^ = 0%).

Subgroup analysis for HHS, survival of implant from aseptic loosening, and radiolucent lines indicated that the included non-RCTs did not affect the RCTs (*p* = 0.42; *p* = 0.27; *p* = 0.98; *p* = 0.52), while as to incidence of thigh pain and femoral osteolysis, there was a slight difference (*p* = 0.02; *p* = 0.05). Our results showed that the longer duration of follow-up tends to have higher HHS (*p* = 0.11) than the shorter one (WMD = 2.21, 95% CI 1.05 to 3.37 for duration of follow-up > 6 years; WMD = 0.58, 95% CI −1.04 to 2.20 for duration < 6 years) and likewise for the survival (*p* = 0.11, RR = 1.01 95% CI 0.99 to 1.03 for duration of follow-up > 6 years, RR = 1.00, 95% CI 0.99 to 1.00 for duration of follow-up < 6 years). The thickness of HA was larger than 80 μm or less than 50 μm, the purity less than 90%, and the anatomic implant reduced incidence of thigh pain and the duration of follow-up did not affect it. It is doubtful that the RCTs and high-quality study had the high incidence of thigh pain and femoral osteolysis (Table 4). The overall result was not significantly altered by omitting trials with a sample sizes less than 70 or those with imputed data.

**Table 4:** **Subgroup analysis of the included studies by different influential factors**

**Factors**	**Harris hip score**		**Survival**		**Incidence of thigh pain**		**Radiolucent lines**		**Femoral osteolysis**		**Polyethylene wear**	
	**Subgroups (numbers)**	**WMD (95% CI)**	**Subgroups (numbers)**	**RR (95% CI)**	**Subgroups (numbers)**	**OR (95% CI)**	**Subgroups (numbers)**	**OR (95% CI)**	**Subgroups (numbers)**	**OR (95% CI)**	**Subgroups (numbers)**	**WMD (95% CI)**
Study design	RCT (10)	1.41 (0.30, 2.52)	RCT (10)	1.01 (0.99, 1.02)	RCT (4)	0.73 (0.42, 1.28)	RCT (4)	0.95 (0.65, 1.39)	RCT (2)	1.37 (0.45, 4.17)	RCT (4)	N.A.
	Non-RCT (5)	2.29 (0.50, 4.07)	Non-RCT (6)	1.00 (0.99, 1.00)	Non-RCT (2)	0.14 (0.04, 0.49)	Non-RCT (2)	0.96 (0.41, 2.24)	Non-RCT (3)	0.39 (0.22, 0.70)	Non-RCT (0)	
		*p* = 0.42		*p* = 0.27		*p = 0.02*		*p* = 0.98		*p = 0.05*		
Study quality	High (9)	1.41 (0.30, 2.52)	High (8)	1.01 (0.99, 1.02)	High (3)	1.29 (0.57, 2.88)	High (4)	0.95 (0.65, 1.39)	High (2)	1.24 (0.38, 4.13)	High (4)	N.A.
	Moderate (6)	2.28 (0.50, 4.07)	Moderate (8)	1.00 (0.99, 1.00)	Moderate (3)	0.30 (0.15, 0.57)	Moderate (2)	0.96 (0.41, 2.24)	Moderate (2)	0.42 (0.24, 0.74)	Moderate (0)	
		*p* = 0.42		*p* = 0.27		*p = 0.006*		*p* = 0.98		*p* = 0.11		
Thickness of HA	50–80 μm (8)	0.76 (−0.63, 2.15)	50–80 μm (10)	1.00 (0.99, 1.00)	50–80 μm (3)	0.71 (0.40, 1.27)	50–80 μm (5)	N.A.	50–80 μm (3)	N.A.	50–80 μm (2)	0.00 (−0.01, 0.01)
	<50 μm or >80 μm (3)	2.03 (0.41, 3.64)	<50 μm or >80 μm (4)	1.02 (0.98, 1.06)	<50 μm or >80 μm (2)	0.19 (0.06, 0.58)	<50 μm or >80 μm (0)		<50 μm or >80 μm (0)		<50 μm or >80 μm (2)	−0.03 (−0.03, −0.03)
		*p* = 0.24		*p* = 0.23		*p = 0.04*						*p < 0.00001*
Purity of HA	>90% (4)	−0.09 (−3.04, 2.86)	>90% (7)	1.00 (0.99, 1.00)	>90% (2)	0.68 (0.36, 1.26)	>90% (3)	0.73 (0.31, 1.71)	>90% (2)	0.66 (0.27, 1.62)	>90% (1)	0.01 (−0.06, 0.08)
	<90% (3)	0.82 (−0.77, 2.42)	<90% (3)	1.00 (0.98, 1.02)	<90% (3)	0.23 (0.08, 0.65)	<90% (2)	0.93 (0.61, 1.40)	<90% (1)	1.24 (0.38, 4.13)	<90% (1)	0.00 (−0.01, 0.01)
		*p* = 0.59		*p* = 0.72		*p* = 0.08		*p* = 0.63		*p* = 0.4		*p* = 0.79
Implant design	Anatomic (5)	1.65 (0.15, 3.16)	Anatomic (4)	1.02 (0.99, 1.05)	Anatomic (2)	0.19 (0.06, 0.58)	Anatomic (1)	0.32 (0.07, 1.47	Anatomic (2)	0.66 (0.27, 1.62)	Anatomic (2)	−0.03 (−0.03, −0.03)
	Non-anatomic (10)	1.66 (0.44, 2.87)	Non-anatomic (12)	1.00 (1.00, 1.00)	Non-anatomic (4)	0.72 (0.41, 1.28)	Non-anatomic (5)	1.01 (0.71, 1.45)	Non-anatomic (3)	0.46 (0.25, 0.85	Non-anatomic (2)	0.00 (−0.01, 0.01)
		*p* = 1.00		*p* = 0.24		*p = 0.04*		*p* = 0.15		*p* = 0.52		*p < 0.00001*
Follow-up duration	>6 years (9)	2.21 (1.05, 3.37)	>6 years (10)	1.01 (0.99, 1.03)	>6 years (4)	0.56 (0.29, 1.08)	>6 years (2)	0.58 (0.24, 1.43)	>6 years (4)	0.44 (0.24, 0.77)	>6 years (3)	−0.02 (−0.02, −0.01)
	<6 years (6)	0.58 (−1.04, 2.20)	<6 years (6)	1.00 (0.99, 1.00)	<6 years (2)	0.50 (0.24, 1.05)	<6 years (4)	1.04 (0.71, 1.52)	<6 years (1)	1.00 (0.32, 3.15)	<6 years (1)	0.01 (−0.06, 0.08)
		*p* = 0.11		*p* = 0.11		*p* = 0.82		*p* = 0.25		*p* = 0.2		*p* = 0.46

Study design and study quality would affect the incidence of thigh pain and study design has influence on femoral osteolysis. When the thickness of HA is <50 or >80 μm, it has less thigh pain incidence and polyethylene wear. The anatomic implant has less incidence of thigh pain and polyethylene wear.

*WMD* weighted mean difference, *N.A.* not available. *HA* hydroxyapatite.

## Discussion

The primary finding is that HA coating could improve the postoperative HHS, reduce the incidence of thigh pain, and reduce the incidence of femoral osteolysis while there was no statistical difference of femoral stem survivorship from aseptic loosening, polyethylene wear, and radiolucent lines between the two groups. In addition, the subgroup analyses found that HHS tends to improve in the longer duration of follow-up and so was the prosthetic survival. The longer duration of follow-up, the better advantage of HA coating over porous coating for the HHS and survivorship from aseptic loosening.

In this meta-analysis, we asked: (1) which coating is better with regard to the clinical and radiologic measurements and (2) which modifying factors affect the comparative effect between both coatings.

To the best of our knowledge, the present meta-analysis is the first to comprise all the available comparative observational evidence and to comprehensively investigate the difference in HHS and survivorship and radiographic outcomes between HA and porous coating for THA. As the previous systematic review only included four RCTs of nine studies, one of the included studies did not show clear HHS data and the HA stem was grit-blasted, while porous stem was not. Another previous systematic review did not include new studies published in the later years. We included 12 RCTs and 9 comparison observation studies and developed explicit inclusion and exclusion criteria. Our analysis quintupled the sample size compared with previous meta-analysis (9,860 versus 1,764) and had longer duration of follow-up (7.5 years versus 6.5 years). We performed a comprehensive set of subgroup analyses and a sensitivity analysis not only to explain the heterogeneity but also to provide additional insights into the potential influential factors of HHS, survival, thigh pain, and other radiographic outcome.

Our meta-analysis has some limitations. (1) There was variability in the selection criteria of individual trials, including the primary disease, gender, ages of patients, and the type of prosthesis. (2) due to the limited number of included trials, we could not analyze the influence of other clinically relevant factors, such as complications of THA, BMD, and WOMAC osteoarthritis index. (3) Missing information such as declining participation and crossover led to incomplete data and potentially bias. (4) The small sample size in the subgroup analysis reduced the precision of the pooled estimates and the ability to detect the statistical significance of some variables, that is, polyethylene wear. More RCTs would be warranted to clarify them. (5) With the limitation of included studies, we can not analyze the effect of implant design, which needs more study to assess.

One of the most significant results of our analysis is that HA coating had higher HHS and less incidence of thigh pain. The advantage of an HA coating includes superior proximal femoral osteointegration and better preservation of periprosthetic bone quality. The patients with HA-coated stems demonstrated significantly lower incidence of activity-related trochanteric and thigh pain [35]. The thickness and purity of HA and implant design could affect the incidence of thigh pain. The incidence decreased abruptly after the first postoperative year [40]. Thigh pain following uncemented hip arthroplasty was generally transient and would disappear over time.

Femoral osteolysis and polyethylene wear could result to gradual subsidence or loosening of implant. Some studies showed that HA coating had less osteolysis and polyethylene wear [37,16], but in Almeida’s study, with the use of HA coated stem, they still found 38% of osteolysis and 41% of polyethylene wear in the hips. Their stems were mostly used in young patients, who had greater activity compared to the older patients which might have been the affecting factor. From the retrieved specimen, some studies found that HA coating could increase the amount of ingrowth and attachment of bone leading to the enhanced biological fixation [41]. Moreover, HA-coated Ti implants can achieve a much higher degree of bone apposition and mechanical stability compared to the implants without such a coating [26]. The heterogeneity of polyethylene wear can not be explained by purity of HA and duration of follow-up. But when we classified the polyethylene wear into subgroups by the thickness of HA (50–80 μm and >80 μm or < 50 μm) and implant design (anatomic and non-anatomic), the heterogeneity could no longer be observed.

The geometry design of the implant has a large impact on the clinical outcome. Joshi’s study explored the hypothesis that through redesign, a total hip prosthesis could be developed to substantially reduce stress shielding, then reduce the loosening of the prosthesis [42]. Dopico-González assessed effects of implant design geometry by probabilistic finite element tool, she thought the geometry of the implant design clearly affected the sensitivities of maximum nodal micromotion [43]. As the limitation of included studies, we just divided the implant into anatomic and non-anatomic. It needs more studies to assess the effect of implant design. Our study showed that anatomic implant had less incidence of thigh pain and reduced polyethylene wear. Ando et al. found that FMS-anatomic stem reduced the proximal stem-bone relative motion and transferred more load to the proximal femur compared to conventional symmetric stems and the FMS, which resulted in better biomechanical stability at least in the early postoperative period [44]. Another study showed an excellent clinical outcome and 98.3% survival of ABG II implant with HA coating [45]. In Cao’s study, they also thought that an anatomically designed prosthesis can provide good clinical results, with low incidence of thigh pain and loosening of the component [46].

HA is biocompatible and osteoconductive and in contact with bone often develops a mechanically tight bond. Human retrieval studies have shown that HA-coated stem observed significantly more ingrowth and attachment of the bone [47].

## Conclusion

In conclusion, HA is better than porous coating. HA coating could improve the postoperative HHS, reduce the incidence of thigh pain, and reduce the incidence of femoral osteolysis, while there was no statistical difference of femoral stem survivorship from aseptic loosening, polyethylene wear, and radiolucent lines between the two groups.

## Abbreviations

HA: hydroxyapatite
HHS: Harris hip score
THA: total hip arthroplasty
RCT: randomized controlled trial
WMD: weighed mean difference
CI: confidence intervals
RR: relative risk
BMD: bone mineral density
WOMAC: Western Ontario and McMaster Universities Arthritis Index
